# Post‐fire pickings: Large herbivores alter understory vegetation communities in a coastal eucalypt forest

**DOI:** 10.1002/ece3.8828

**Published:** 2022-04-23

**Authors:** Matthew Chard, Claire N. Foster, David B. Lindenmayer, Geoffrey J. Cary, Christopher I. MacGregor, Wade Blanchard

**Affiliations:** ^1^ 590680 Fenner School of Environment & Society The Australian National University Canberra ACT Australia; ^2^ 590680 Threatened Species Recovery Hub National Environmental Science Program Fenner School of Environment & Society The Australian National University Canberra ACT Australia

**Keywords:** herbivory, large herbivore, macropod, post‐fire, vegetation community

## Abstract

Fire and herbivores alter vegetation structure and function. Future fire activity is predicted to increase, and quantifying changes in vegetation communities arising from post‐fire herbivory is needed to better manage natural environments. We investigated the effects of post‐fire herbivory on understory plant communities in a coastal eucalypt forest in southeastern Australia. We quantified herbivore activity, understory plant diversity, and dominant plant morphology following a wildfire in 2017 using two sizes of exclosures. Statistical analysis incorporated the effect of exclusion treatments, time since fire, and the effect of a previous prescribed burn. Exclusion treatments altered herbivore activity, but time since fire did not. Herbivory reduced plant species richness, diversity, and evenness and promoted the dominance of the most abundant plants within the understory. Increasing time since fire reduced community diversity and evenness and influenced morphological changes to the dominant understory plant species, increasing size and dead material while decreasing abundance. We found the legacy effects of a previous prescribed burn had no effect on herbivores or vegetation within our study. Foraging by large herbivores resulted in a depauperate vegetation community. As post‐fire herbivory can alter vegetation communities, we postulate that management burning practices may exacerbate herbivore impacts. Future fire management strategies to minimize herbivore‐mediated alterations to understory vegetation could include aggregating management burns into larger fire sizes or linking fire management with herbivore management. Restricting herbivore access following fire (planned or otherwise) can encourage a more diverse and species‐rich understory plant community. Future research should aim to determine how vegetation change from post‐fire herbivory contributes to future fire risk.

## Graphical abstract

We investigated the effects of post‐fire herbivory on understory plant communities in a coastal eucalypt forest in southeastern Australia. We found foraging by large herbivores resulted in a depauperate vegetation community. As post‐fire herbivory can alter vegetation communities, we postulate that management burning practices may exacerbate large herbivore impacts and these impacts must be considered in the future.

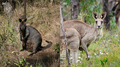

## 1 INTRODUCTION

Fire and herbivores are consumers of vegetation, modifying the structure and function of plant communities (Bond & Keeley, 2005). Interactions between these disturbances can occur in ecosystems where both fire and herbivores are prevalent. However, few studies have implemented manipulative field studies to measure the long‐term responses of forest vegetation to both fire and herbivory (Foster et al., 2016; Nuttle et al., 2013; Royo & Carson, 2006). As forest ecosystems are likely to face increased fire activity in the future (Bowman et al., 2009), more research that quantifies interactions between fire and herbivory on plant communities is required.

**FIGURE 1:**
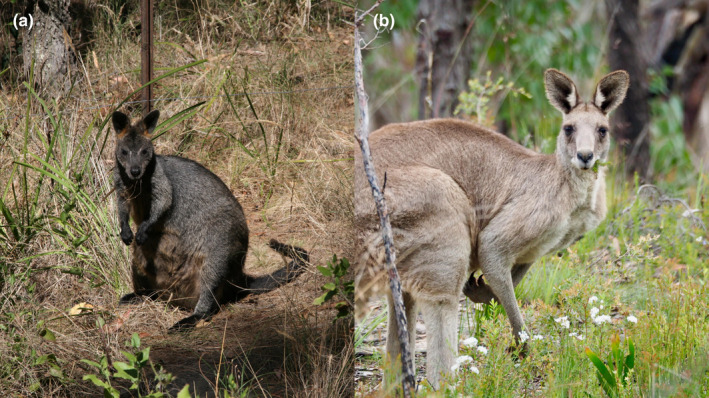
Large herbivores present in our study, the (a) swamp wallaby (*Wallabia bicolor*) and the (b) eastern grey kangaroo (*Macropus giganteus*). Photo credit: C. N. Foster & J. Clarke

Fire can encourage or deter herbivore foraging (Allred et al., 2011; Fuhlendorf et al., 2010). Research into fire–herbivore relationships, under the banner of “pyric herbivory,” has emphasized the capacity for fire to influence foraging selection by herbivores (Allred et al., 2011). Large herbivores (>2 kg) can be attracted to burnt patches due to increased abundance of new growth and more favorable physical or chemical accessibility to food (Allred et al., 2011; Danell et al., 2006; Foster et al., 2016). However, limited research has been conducted on the next logical question within fire‐prone ecosystems, which is: How does the vegetation community respond when it is burnt, and then foraged? Manipulative experiments are required to answer this question (Figure 1).

**FIGURE 2:**
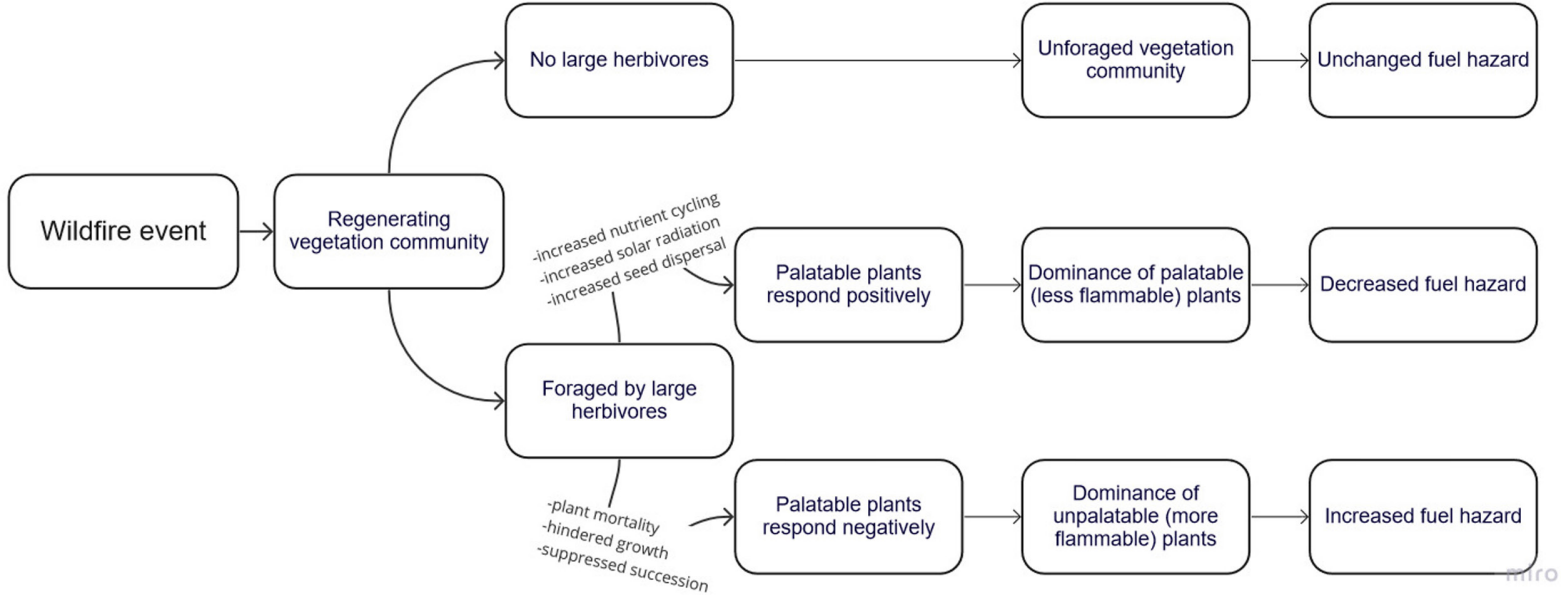
Hypothesised outcomes of foraging from large herbivores following a fire event in a eucalypt forest environment

Information on changes in vegetation communities arising from post‐fire herbivory may guide future fire management practices. Large herbivores can alter plant succession following a fire through foraging, trampling, and alterations in nutrients (by defecation, urination, decomposition of carcasses, etc.; Forbes et al., 2019; Persson et al., 2000). Herbivores actively select for more palatable species, which leads to the dominance of unpalatable, chemically defended plant species, or an increased abundance of highly palatable plants through nutrient cycling and seed dispersal (Augustine & McNaughton, 1998; Bakker et al., 2016; Leroux et al., 2020). Changes to aboveground plant biomass is a direct modification of *in situ* fuel load (Archibald & Hempson, 2016). Fire also can promote the abundance of more flammable plants (through positive feedback loops) that are often less palatable for herbivores (due to lower moisture content, increased tannins/oils, higher carbon‐nitrogen ratios, etc.; Archibald & Hempson, 2016). As dominant plant species can influence fire risk (Cheney et al., 2012; Zylstra et al., 2016), quantifying the changes to plant communities from post‐fire herbivory will be paramount to future wildfire management and predictions (Figure 2).

We aimed to answer the question: How does post‐fire herbivory alter understory plant communities and physical attributes of the dominant plants in a eucalypt forest? We used a manipulative field study at Booderee National Park that modified large herbivore activity through different levels of fencing. At all plots, we tested the effects of fencing treatments and time since fire on herbivore activity, vegetation community measures, and morphological responses of the dominant understory plant. Where applicable, we also investigated the effect of the previous prescribed burn (5 years prior) applied to selected plots. We expected large herbivores to be attracted to recently burnt areas due to the availability of fresh growth and herbivore occurrence would decline over time as the vegetation regenerates (Allred et al., 2011). This response has been observed following prescribed burns in eucalypt forest (Foster et al., 2015; Parkins et al., 2019). However, it is possible that this effect will be less evident following a larger wildfire where herbivores have a larger area of burnt space to select from.

While fire can reduce habitat complexity (Parkins et al., 2019), the interaction of fire and (increased) herbivory may result in an altered vegetation community with reduced species diversity (Foster et al., 2015). Fire will promote germination and growth of understory plants, resulting in a short‐term increase in species richness (Ross et al., 2002). Species richness within the understory plant community typically decreases with time since fire due to increased competition and reduced space (Foster et al., 2018). Large herbivores can dramatically alter the recovering understory vegetative community by preferentially selecting the more palatable species (Persson et al., 2000). We expect that this should result in a decrease in community measures such as richness, diversity, and evenness and promote the dominance of less palatable plants within burnt patches (Foster et al., 2016). In addition, we expect the influence of the prior prescribed burn to exacerbate herbivore impacts following a wildfire as the vegetation has been subject to a short (5 years) fire interval and may be more sensitive to further disturbance compared to areas that were not subject to the prescribed burn (Furlaud et al., 2018).

A particular concern for managers of our study area has been the increase in dominance of the fern *Pteridium esculentum* (bracken) in the understory vegetation (Dexter et al., 2013). Current management of Booderee National Park is based on the understanding that abundant large herbivore populations, coupled with recurrent fires (prescribed burns and wildfires), are promoting bracken dominance (Dexter et al., 2013). Bracken has reduced palatability for larger herbivores (Di Stefano & Newell, 2008) and is an early‐successional and fire‐resistant plant (Tolhurst & Turvey, 1992). The ramifications for both biodiversity and future fire risk resulting from a bracken‐dominated understory are currently unknown. We aimed to provide quantitative evidence on the effect that post‐fire herbivory has on the morphology and abundance of bracken.

By focusing our study in a post‐wildfire eucalypt forest, we predicted that: (1) exclosure treatments would reduce herbivore activity and herbivore activity would decline with increasing time since fire; (2) increased herbivore activity and increasing time since fire would reduce the species richness, diversity, and evenness of the plant community and increase understory dominance of unpalatable understory species; and (3) increased herbivore activity and increasing time since fire would alter morphological measures of the dominant understory plant, promoting larger plants and higher abundance within plots. Furthermore, we predicted the influence of the previous prescribed burn would exacerbate herbivore impacts. Plots subject to the prescribed burn followed by large herbivore browsing were expected to have lower initial plant diversity prior to the 2017 fire (Foster et al., 2015). Therefore, the 2017 wildfire and subsequent foraging by large herbivores was expected to further decrease plant diversity and increase dominant plant abundance at sites burnt in 2012.

## 2 METHODS

### 2.1 Study area

**FIGURE 3:**
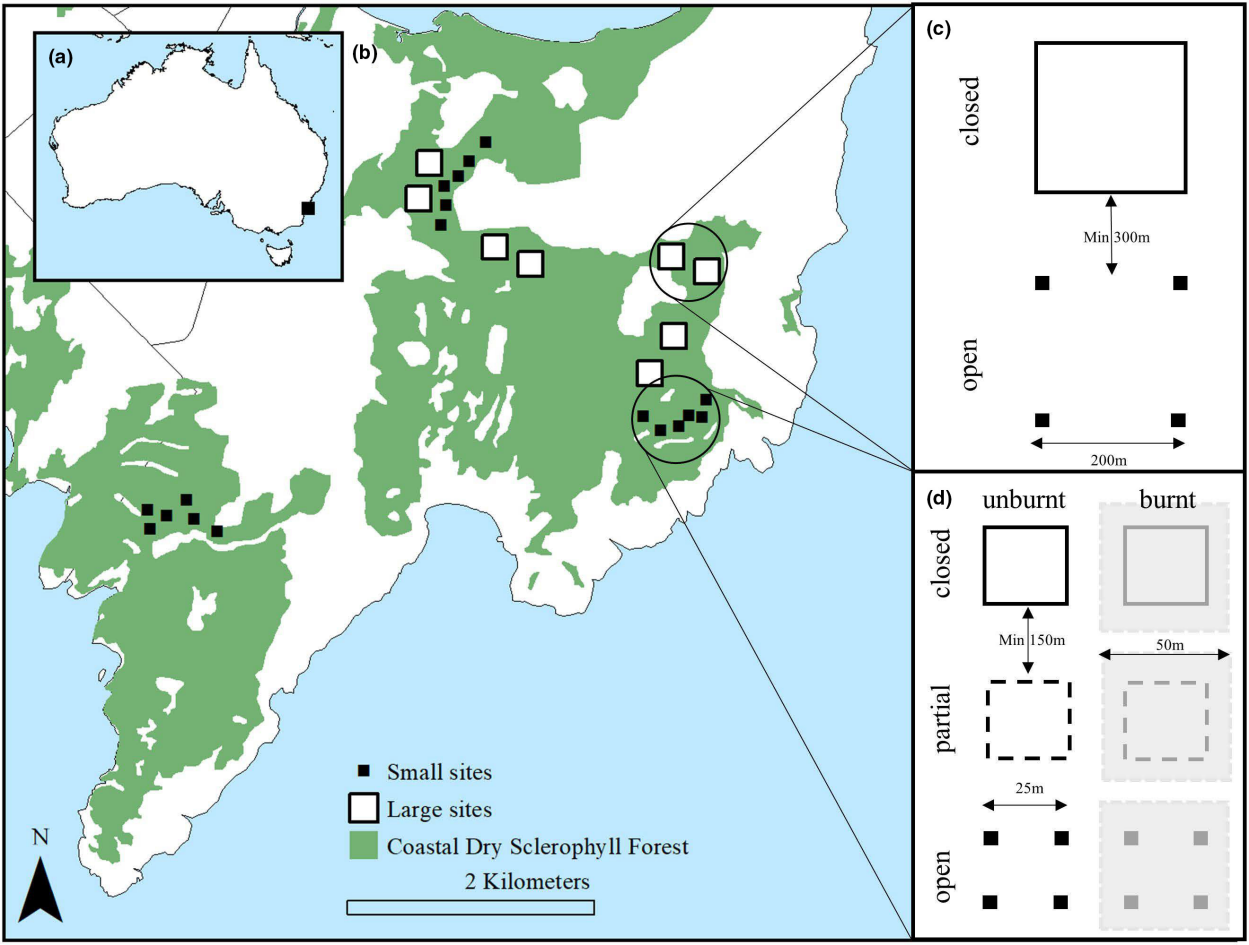
Study location and experimental design showing: (a) Location of Booderee National Park, Australia; and (b) The distribution of the small and large plots across the park. All plots were located within coastal dry sclerophyll forest (in green) and had been burnt in the 2017 wildfire. Eight large plots were paired into four blocks and 18 small plots were grouped into 3 blocks; (c) and (d) highlight the design within each block for both large and small plots respectively. Note half of the small plots were subject to a prescribed burn conducted in 2012

We conducted this study at Booderee National Park (35.1489415°S, 150.6454625°E; Figure 3) on the southeast coast of Australia, approximately 200 km south of Sydney. The Park is ~6500 ha in area and co‐managed by the Wreck Bay Aboriginal Community and Parks Australia. The dominant vegetation class in the park is Sydney Coastal Dry Sclerophyll Forest (45% of the park area) which is characterized by canopy species of *Eucalyptus pilularis*, *Corymbia gummifera*, and *Eucalyptus botryoides*, midstory species of *Banksia serrata* and *Monotoca eliptica*, and an understory dominated by *P*. *esculentum*, *Lomandra longifolia*, and *Lepidosperma concavum* (Taws, 1997).

### 2.2 Study species

Three species of macropod in Booderee National Park meet the “large herbivore” classification (>2 kg; *sensu* Danell et al., 2006). They are the eastern grey kangaroo (*Macropus giganteus*); swamp wallaby (*Wallabia bicolor*); and red‐necked wallaby (*M*. *rufogriseus*). No other large terrestrial herbivore species are currently found in Booderee National Park. All three macropods have previously demonstrated pyric herbivory responses with most studies identifying a preference for recently burnt patches due to a higher quality of foraging resources (Foster et al., 2015; Meers & Adams, 2003; Parkins et al., 2019; Southwell & Jarman, 1987).

Of the three macropod species, the eastern grey kangaroo and the swamp wallaby are the most common in Booderee National Park (Chard et al., 2021). While density measures have not been conducted for any macropod species, previous research analyzing conditional abundance of the swamp wallaby peaked between 2007 and 2013 compared to when surveys began in 2003 (Lindenmayer et al., 2016). Furthermore, managers have been concerned with the growing population of macropods in Booderee National Park (Dexter et al., 2013). Previous research in the study area found both species preferentially selected forest vegetation communities (Chard et al., 2021). The eastern grey kangaroo is a grazing species, targeting grass species such as *Imperata cylindrica* and *Themeda triandra* (Brunton et al., 2018). The swamp wallaby is a browsing species that will forage on most understory plants in a forest community with a preference for forb species (Di Stefano & Newell, 2008). For more detailed descriptions on each species preferred plant foods see Chard et al. (2021).

### 2.3 Study design

We quantified the interacting effects of post‐fire herbivory on vegetation communities using two randomized, blocked experiments. In June 2012, we established three blocks of six 25 × 25 m plots (0.0625 ha, hereafter referred to as “small” plots) within Sydney Coastal Dry Sclerophyll Forest, with plots spaced 150 m apart and blocks 2 km apart. We manipulated grazing pressure by macropods using three methods of fencing: (1) open (i.e., no fencing), (2) partial fencing – intermediate access with gates at two corners of the plot which were opened or closed at 2‐month intervals, and (3) closed (completely fenced). We constructed 1.1‐m‐tall fences which prevented access by macropods (Foster et al., 2015). We conducted low‐intensity, prescribed burns in August 2012 within half of the plots in each block so that each fencing treatment had one burnt and one unburnt pair. Controlled fires were extinguished after burning a 50 × 50 m area and removing approximately 95% of the understory vegetation. This facilitated examination of two burning treatments across three herbivory measures within all three blocks. Although a wildfire in 2017 burnt all plots, we describe our small plots as “burnt” or “unburnt” as per the initial prescribed burn conducted in 2012.

In September 2017, a wildfire burned 1600 ha of Booderee National Park, including each small experimental block, again removing approximately 95% of the understory vegetation. In our study, we recorded time since fire as time since the 2017 wildfire. In July 2018, we established an additional four blocks of two 200 × 200m plots (4 ha, hereafter referred to as “large” plots) in forest vegetation. Large plots were spaced 300 m apart and blocks at least 2 km apart. Again, we manipulated herbivore grazing pressure using two randomly allocated fencing treatments: (1) open (no fencing) and (2) closed (completely fenced).

### 2.4 Data collection

We conducted scat surveys every 2 months from October 2018 to February 2020 in all plots, within two 25 m × 2 m transects (small plots), and four 50 m × 2 m transects (large plots), in which macropod scats were counted and removed from the transect. We used macropod scat counts as an index of herbivore activity, as macropods defecate primarily while feeding (Johnson et al., 1987; Murphy & Bowman, 2007). Note, it was assumed in this study that macropods will digest and deposit all vegetation at a similar rate. We conducted vegetation surveys annually in spring, in all plots. We used five point‐intercept transects of 20 m (small plots) and four 50 m transects (large plots) within each plot to record understory plant species (<3 m in height) at 1 m intervals. We used site‐level data to calculate four vegetation community measures: species richness, diversity (Simpson's reciprocal index – 1/*D*), evenness (Shannon evenness index), and dominance (Berger–Parker index; Magurran, 2013). Using the same point‐intercept transects, when a bracken plant was present, we recorded its physical attributes including width (measured parallel with the transect), height to bottom‐most frond, top height, and percentage of dead vegetation. We also recorded the number of bracken plants intercepted at the 20 or 50 points along each transect. Both scat and vegetation surveys encompassed the post‐wildfire period from September 2018 to February 2020.

### 2.5 Data analysis

We analyzed the influence of exclosure fences, time since fire, and the 2012 prescribed burn on: (1) scat counts, (2) plant community measures, and (3) bracken attributes in R (R Core Team, 2016). We fit models from a candidate set of nine models (small plots) and two models (large plots) for each response in a Bayesian framework using the “brms” package (Bürkner, 2017). The models we constructed used all possible combinations of exclusion treatment (open/partial/closed), time since fire, and prescribed burn (burnt/unburnt) for each response variable (Tables S1–S3). We selected appropriate regression distributions for each variable after testing for assumptions of normality and homogeneity of variance (see Tables S1; Tables 2 and 3; Hanea et al., 2015).

Our response variables were as follows: (1) number of macropod scats, with scat counts being summed at 2‐month intervals for small plots to allow for effective analysis of the partial treatments (as every second count was effectively zero); (2) understory plant richness, diversity, evenness, and dominance, with vegetation measures calculated using the “diversityresult” function from the “BiodiversityR” package (Kindt & Kindt, 2019); and (3) bracken width, height to bottom frond, top height, count of individuals, and percentage of dead material. We treated time since fire as a continuous variable for scat surveys, standardized using the “scale” function so that the mean was zero with a standard deviation of 1. We included season (for scat surveys) and block as a fixed effect in each model as well as the random effect of plot. We expected a seasonal effect resulting in reduced herbivore activity in the summer months as macropod defecation rates decrease and scat decay increases (Perry & Braysher, 1986). We selected appropriate priors for each model and the Rhat values were deemed acceptable (all values = 1; Gelman & Rubin, 1992).

The models were fit using Markov chain Monte Carlo methods. We ran four chains, each with 3000 iterations with the first 1000 iterations discarded as burn‐in for the sampler. We based our inference on the importance of the hypothesized interactions by selecting the most parsimonious model using lowest weighted Akaike information criterion (WAIC; ≤2) and simplest model using the “loo” package (Burnham & Anderson, 2002; Vehtari et al., 2017). We selected AIC over the Bayesian information criterion (BIC) to allow the inclusion of more potential predictors in the model (Aho et al., 2014). We present results for most parsimonious models for macropod scats, vegetation community measures, and bracken morphology from small and large plots.

## 3 RESULTS

### 3.1 Herbivore activity

**FIGURE 4:**
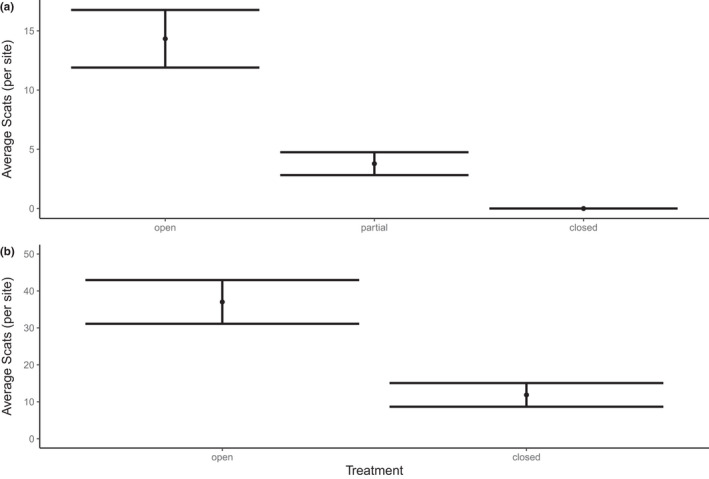
The average abundance of macropod scats found in (a) small plots and (b) large plots. Small plots were 25 m × 25 m and had three levels of fencing (open/closed/partial) to alter macropod access. Large plots were 200 m × 200 m and had two level of fencing (open/closed) to alter macropod access. Values are means and 95% credible intervals from plots located in forest vegetation

Our exclosure treatments altered herbivore activity (Table 1). The best performing model for both small and large plots did not include any interaction terms (Table S1). In both small and large plots, scat counts were highest in the open treatment and lowest in the closed treatments (Figure 4). We found in the small plots that partial treatments had scat counts at intermediate levels between open and closed treatments. Time since fire did not influence herbivore activity in either large or small plots. Furthermore, we detected no effect of the previous prescribed burns in the small plots. Season only affected scat counts within the large plots, with counts being lower in summer months. Notably, scat counts in the large, closed plots were not zero (11.9 ± 6.5 SE), indicating some level of macropod intrusion within the exclosure fences (Figure 4).

**TABLE 1:** Results from Bayesian generalized linear model analyzing whether macropod scats in small (25 m × 25 m) and large (200 m × 200 m) plots are influenced by fire (burnt/unburnt), herbivore access (open/partial/closed), and time since fire

Coefficient	Small plots		Large plots	
	Est.	CI (95%)	Est.	CI (95%)
Intercept	**2.40**	**0.99, 3.98**	**4.33**	**2.61, 5.60**
Herbivory (Partial)	**−1.45**	**−2.38, −0.51**		
Herbivory (Closed)	**−7.76**	**−12.91, −5.31**	**−1.55**	**−2.72, −0.13**
Fire (Burnt)	−0.48	−1.42, 0.43		
Time Since Fire	0.48	−0.09, 1.07	−0.17	−0.46, 0.11
Summer	0.20	−1.11, 1.47	**−1.20**	**−1.98, −0.44**
Autumn	−0.01	−1.61, 1.58	−0.75	−1.61, 0.18
Winter	0.50	−0.98, 2.01	0.32	−0.49, 1.11
Block B	0.16	−0.93, 1.24	−0.07	−1.50, 1.60
Block C	0.45	−0.63, 1.51	−1.22	−2.75, 0.47
Block D			−0.13	−1.63, 1.55
*N*	18_plot_		8_plot_	
Obvs.	126		67	
Marginal *R* ^2^/Conditional *R* ^2^	.474/.492		.589/.629	

Note

Estimates (log‐scale) and 95% credible intervals are shown for the most parsimonious models (by WAIC and model simplicity; see Table S1 for model selection table). Rows that are in bold indicate that credible intervals do not overlap zero. Reference states for comparisons in small plots were open, unburnt plots sampled in 2018. Reference states for comparisons in large plots were open plots sampled in 2018.

### 3.2 Vegetation community

**FIGURE 5:**
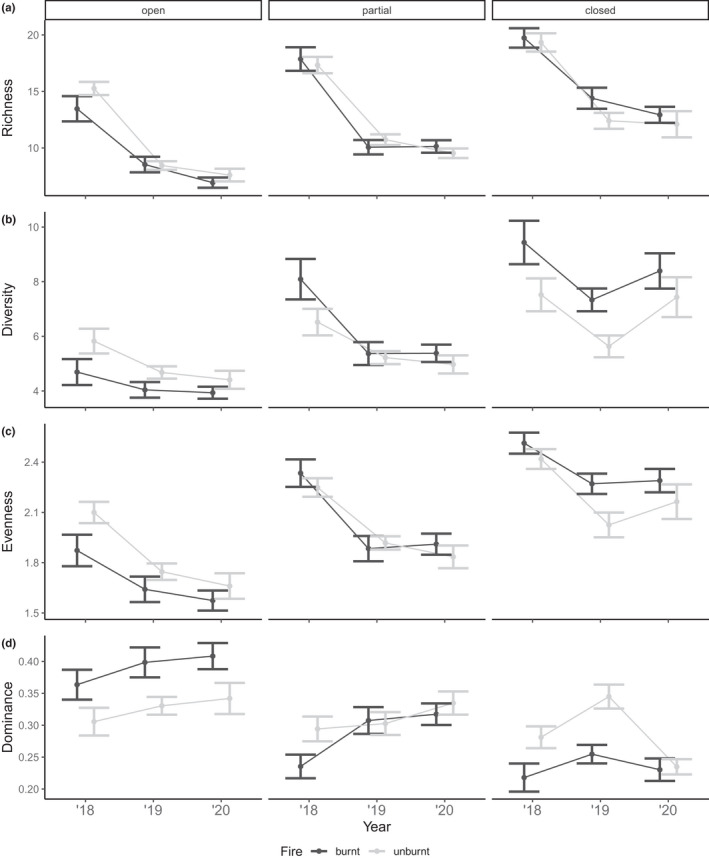
Response of plant community measures of (a) species richness, (b) diversity (Simpson's reciprocal index, 1/*D*), (c) evenness (Simpson's evenness, *E*1/*D*) and (d) dominance (Berger‐Parker, d) to fire (unburnt/burnt) and herbivory (open/partial/closed) through time. Values are means and 95% credible intervals from small plots (25 m × 25 m) in forest vegetation

**FIGURE 6:**
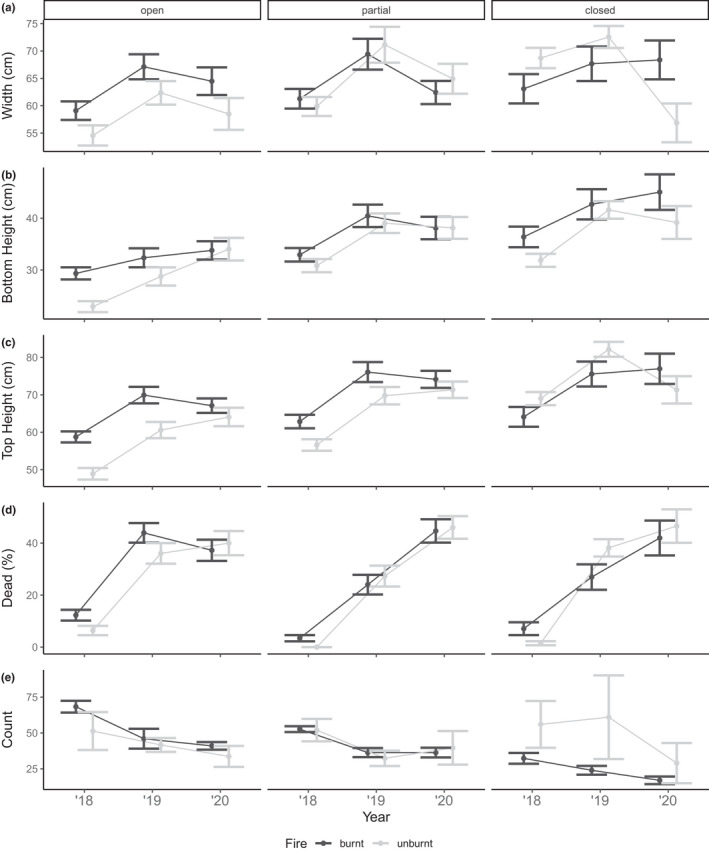
Morphological responses of the dominant understory bracken (*Pteridium esculentum*) of (a) width, (b) height to bottom frond, (c) top height, (d) percent of dead material and (e) number of plants to fire (unburnt/burnt) and herbivory (open/partial/closed) through time. Values are means and 95% credible intervals from small plots (25 m × 25 m) located in forest vegetation

Three years of vegetation surveys yielded 74 plant species in the understory community. We found herbivore activity and time since fire altered understory community measures in both small and large plots (Figures 5 and 6).

In small plots, the interaction of herbivory and time since fire was included in the selected models for diversity and dominance (Table S1). However, the confidence intervals for both interaction effects overlapped with zero indicating a weak effect (Table 2). Richness, diversity, and evenness measures in small, open plots were lower compared to partially closed and closed plots (Figure 5a–c). As time since fire increased, community measures in small, open plots revealed a decreasing trend for richness, diversity, and evenness. This trend was similar in partially closed plots with 2018 richness, diversity, and evenness measures higher compared to 2019 and 2020 surveys. However, in small, closed plots where macropods were fully excluded, diversity and evenness measures were higher in 2020 compared to 2019. In small plots, there was a greater proportion of dominant species present in open plots compared to partially closed and closed plots (Figure 5d). As time since fire increased, dominance measures in small plots that were open and partially closed steadily increased, with 2018 measures being lower compared to 2019 and 2020. Again, small plots that were macropod free were characterized by an initial increase in dominance measures from 2018 to 2019, but then a decrease in 2020.

**TABLE 2:** Results from Bayesian generalized linear model analyzing whether plant species richness, diversity (Simpson's reciprocal index, 1/*D*), evenness (Simpson's evenness, *E*_1/_ *_D_*), and dominance (Berger‐Parker, *d*) in 25 m × 25 m plots are influenced by fire (burnt/unburnt), herbivore access (open/partial/closed), and year (2018/19/20)

Coefficient	Species Richness		Simpson's diversity (1/*D*)		Shannon's evenness (*E*_1/_ *_D_*)		Berger–Parker dominance (*d*)	
	Est.	CI (95%)	Est.	CI (95%)	Est.	CI (95%)	Est.	CI (95%)
Intercept	**2.57**	**2.39, 2.75**	**1.5**	**1.28, 1.71**	**1.93**	**1.75, 2.11**	**0.36**	**0.30, 0.42**
Herbivory (partial)	**0.23**	**0.05, 0.39**	**0.33**	**0.11, 0.56**	**0.25**	**0.08, 0.43**	**−0.07**	**−0.13, −0.01**
Herbivory (closed)	**0.41**	**0.24, 0.57**	**0.49**	**0.26, 0.71**	**0.5**	**0.32, 0.67**	**−0.08**	**−0.15, −0.02**
Fire (burnt)	0	−0.14, 0.14	0.04	−0.12, 0.21	0.02	−0.12, 0.17	−0.01	−0.05, 0.04
Year (2019)	**−0.47**	**−0.55, −0.40**	**−0.19**	**−0.31, −0.06**	**−0.35**	**−0.41, −0.28**	**0.04**	**0.01, 0.07**
Year (2020)	**−0.55**	**−0.63, −0.46**	**−0.16**	**−0.28, −0.02**	**−0.33**	**−0.39, −0.27**	0.03	−0.00, 0.07
Block B	−0.05	−0.22, 0.12	−0.04	−0.23, 0.17	−0.06	−0.24, 0.13	0	−0.06, 0.05
Block C	0.15	−0.02, 0.31	**0.28**	**0.09, 0.48**	**0.24**	**0.07, 0.42**	**−0.07**	**−0.13, −0.01**
Partial: 2019			−0.11	−0.28, 0.06			0	−0.04, 0.05
Closed: 2019			−0.08	−0.25, 0.08			0.02	−0.03, 0.06
Partial: 2020			−0.17	−0.34, 0.01			0.03	−0.02, 0.08
Closed:2020			0.09	−0.08, 0.26			−0.04	−0.09, 0.01
*N*	18_plot_		18_plot_		18_plot_		18_plot_	
Observations	270		270		270		270	
Marginal *R* ^2^/Conditional *R* ^2^	.683/.745		.527/.623		.597/.675		.402/.525	

Note

Estimates and 95% credible intervals are shown for the most parsimonious models (by WAIC and model simplicity; see Table S2 for model selection table). Rows that are in bold indicate that credible intervals do not overlap zero. Reference states for comparisons were open, unburnt plots sampled in 2018.

In large plots, the interaction of herbivory and time since fire was included in richness, diversity, and evenness models (Table S2). The confidence intervals for the interaction effect for species richness overlapped with zero (Table 3). We observed comparable trends to small plots within large plots, with richness, diversity, and evenness measures being lower in open plots compared to closed (Figure 6a–c). For large, open plots, increasing time since fire negatively affected richness, diversity, and evenness, with the highest measures observed in 2018 which subsequently decreased in 2019 and 2020. A different trend for time since fire was apparent for large, closed plots, whereby measures of richness, diversity, and evenness initially decreased from 2018 to 2019, but then increased in 2020. Dominance measures within large plots were higher in open plots, although confidence intervals overlapped with zero. We found time since fire to influence community dominance with measures being higher in 2019 compared to both 2018 and 2020 (Figure 6d). Large, open plots revealed an increasing trend for dominance as time since fire increased, while dominance in closed plots peaked in 2019 before decreasing the following year.

**TABLE 3:** Results from Bayesian generalized linear model analyzing whether plant species richness, diversity (Simpson's reciprocal index, 1/*D*), evenness (Simpson's evenness, *E*_1/_ *_D_*), and dominance (Berger‐Parker, *d*) in 200 m × 200 m plots are influenced by herbivore access (open/partial/closed) and year (2018/19/20)

Coefficient	Species richness		Simpson's diversity (1/*D*)		Shannon's evenness (*E*_1/_ *_D_*)		Berger–Parker dominance (*d*)	
	Est.	CI (95%)	Est.	CI (95%)	Est.	CI (95%)	Est.	CI (95%)
Intercept	**3.01**	**2.81, 3.20**	**2.87**	**2.70, 3.02**	**2.94**	**2.76, 3.10**	**0.09**	**0.06, 0.11**
Herbivory (Closed)	0.11	−0.10, 0.32	0.13	−0.03, 0.29	0.12	−0.04, 0.29	−0.01	−0.03, 0.01
Year (2019)	**−0.21**	**−0.40, −0.03**	**−0.21**	**−0.33, −0.10**	**−0.21**	**−0.33, −0.10**	**0.02**	**0.01, 0.04**
Year (2020)	**−0.3**	**−0.49, −0.11**	**−0.26**	**−0.37, −0.15**	**−0.29**	**−0.40, −0.18**	0.01	0.00, 0.03
Block B	**−0.31**	**−0.55, −0.09**	**−0.28**	**−0.48, −0.10**	**−0.30**	**−0.48, −0.09**	0.02	−0.01, 0.05
Block C	**−0.49**	**−0.73, −0.25**	**−0.47**	**−0.66, −0.28**	**−0.49**	**−0.69, −0.28**	**0.05**	**0.02, 0.07**
Block D	**−0.31**	**−0.54, −0.07**	**−0.28**	**−0.45, −0.10**	**−0.29**	**−0.50, −0.09**	0.02	−0.00, 0.05
Closed:2019	−0.06	−0.32, 0.19	−0.06	−0.23, 0.10	−0.06	−0.22, 0.10		
Closed:2020	0.22	−0.03, 0.47	**0.19**	**0.03, 0.35**	**0.22**	**0.05, 0.38**		
*N*	8_plot_		8_plot_		8_plot_		8_plot_	
Observations	96		96		96		96	
Marginal *R* ^2^/Conditional *R* ^2^	.689/.690		.655/.662		.669/.677		.371/.370	

Note

Estimates and 95% credible intervals are shown for the most parsimonious models (by WAIC and model simplicity; see Table S2 for model selection table). Rows that are in bold indicate that credible intervals do not overlap zero. Reference states for comparisons were open plots sampled in 2018.

Comparison of experimental blocks for small plots revealed Block C to have significantly higher measures of species diversity and evenness and lower measures of dominance compared to Blocks A and B (Table 2). Similarly, in the large plots, Block A supported significantly higher species richness, diversity, and evenness compared to the other three blocks (Table 3). However, Block C had the highest values for plant dominance.

### 3.3 Dominant plant morphology

**FIGURE 7:**
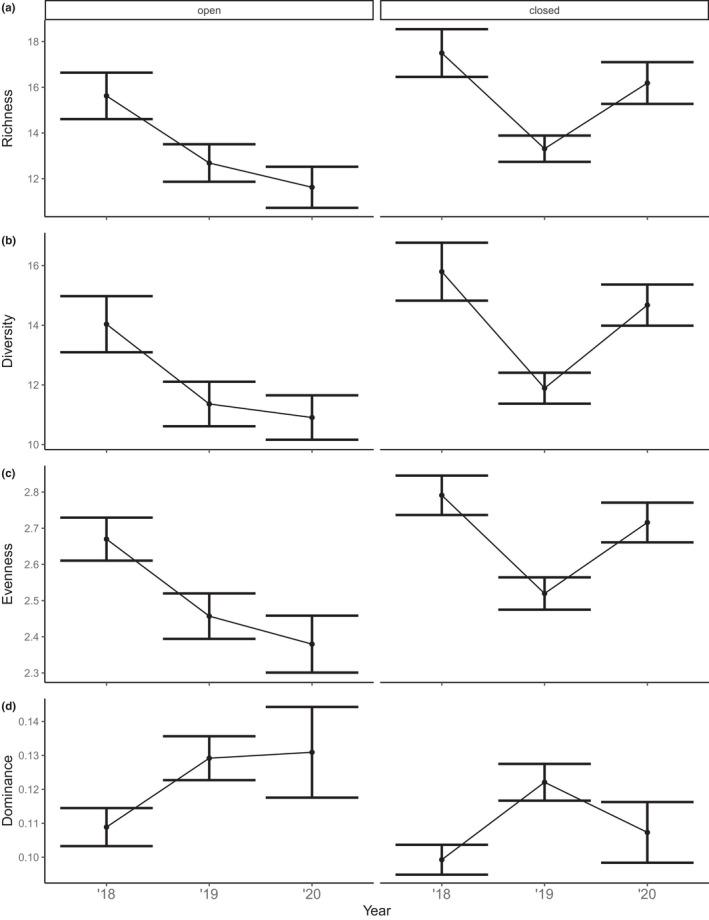
Response of plant community measures of (a) species richness, (b) diversity (Simpson's reciprocal index, 1/*D*), (c) evenness (Simpson's evenness, *E*1/*D*) and (d) dominance (Berger‐ Parker, d) herbivory (open/closed) through time. Values are means and 95% credible intervals from large plots (200 m × 200 m) located in forest vegetation

**FIGURE 8:**
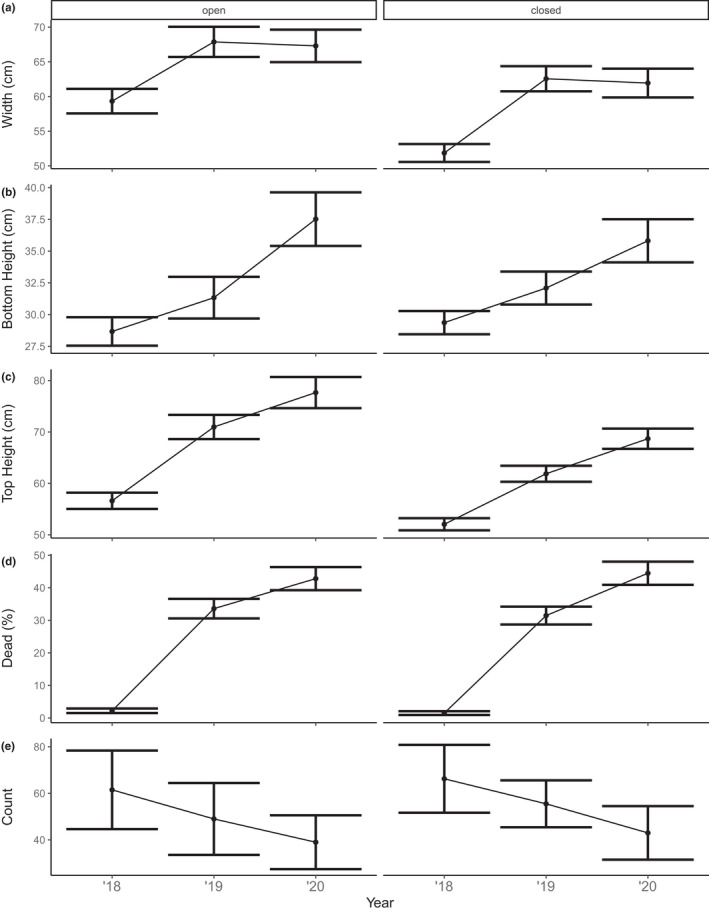
Morphological responses of the dominant understory bracken (*Pteridium esculentum*) of (a) width, (b) height to bottom frond, (c) top height, (d) percent of dead material and (e) number of plants to herbivory (open/closed) through time. Values are means and 95% credible intervals from large plots (200 m × 200 m) located in forest vegetation

During the 3 years of vegetation surveys, we measured 3468 individual *P*. *esculentum* plants. The interaction effect of herbivore activity and time since fire was included only in models for bottom height and for the count of individuals for small plots (Table S3). In small plots, the bottom heights of bracken plants were higher within partial and closed plots in 2019 compared to both 2018 and 2020 (Figure 7b). The number of bracken plants within small plots decreased with time since fire and they were more abundant within closed plots in 2019 (Figure 7e; Table 4). Within large plots, we found bracken width to be altered by herbivore activity, with plants in open plots being wider (Figure 8a).

**TABLE 4:** Results from Bayesian generalized linear model analyzing whether *Pteridium esculentum* (bracken) morphology including: plant width, height to bottom frond, height to top, percentage of dead material on each plant, and number of plants in 25 m × 25 m plots, are influenced by fire (burnt/unburnt), herbivore access (open/partial/closed), and year (2018/19/20)

Coefficient	Width (cm)		Bottom Height (cm)		Top Height (cm)		Dead (%)		Count	
	Est.	CI (95%)	Est.	CI (95%)	Est.	CI (95%)	Est.	CI (95%)	Est.	CI (95%)
Intercept	**60.23**	**55.30, 65.30**	**28.01**	**23.60, 33.11**	**58.96**	**52.33, 65.21**	**0.45**	**0.07, 0.86**	**4.12**	**3.65, 4.59**
Herbivory (partial)	0.55	−3.80, 4.80	1.02	−3.00, 5.28	0.24	−4.75, 4.80	−0.27	−0.57, 0.01	−0.12	−0.61, 0.37
Herbivory (closed)	2.03	−2.11, 6.81	2.87	−1.62, 7.70	2.06	−2.73, 8.20	−0.07	−0.40, 0.27	−0.42	−0.91, 0.10
Fire (burnt)	−0.23	−4.20, 3.74	0.77	−3.07, 4.55	0.40	−4.18, 5.24	0.16	−0.09, 0.41	0.11	−0.28, 0.49
Year (2019)	**5.74**	**3.32, 8.28**	**3.35**	**0.80, 5.75**	**10.42**	**8.17, 12.68**	**−0.43**	**−0.76, −0.11**	**−0.31**	**−0.47, −0.16**
Year (2020)	1.17	−1.23, 3.61	**5.68**	**3.25, 8.28**	**10.29**	**7.91, 12.68**	**−0.59**	**−0.93, −0.24**	**−0.47**	**−0.63, −0.30**
Block B	0.68	−3.38, 4.92	0.75	−3.48, 4.86	0.15	−4.59, 5.25	−0.13	−0.42, 0.18	0.04	−0.43, 0.51
Block C	1.58	−2.41, 6.09	3.17	−1.19, 8.36	1.11	−3.53, 6.34	−0.29	−0.65, 0.05	−0.42	−0.88, 0.04
Partial: 2019			**3.54**	**0.26, 7.27**					−0.11	−0.35, 0.12
Closed: 2019			**4.26**	**0.93, 8.16**					**0.27**	**0.04, 0.50**
Partial: 2020			0.09	−3.09, 3.31					0.15	−0.09, 0.38
Closed: 2020			1.28	−2.24, 5.19					0.04	−0.23, 0.32
*N*	18_plot_		18_plot_		18_plot_		18_plot_		18_plot_	
Observations	2210		2210		2210		2210		53	
Marginal *R* ^2^/Conditional *R* ^2^	.018/.061		.046/.112		.053/.189		.001/.001		.084/.733	

Note

Estimates and 95% credible intervals are shown for the most parsimonious models (by WAIC and model simplicity; see Table S2 for model selection table). Rows that are in bold indicate that credible intervals do not overlap zero. Reference states for comparisons were open, unburnt plots sampled in 2018.

As time since fire increased, bracken plants were wider and taller within both small and large plots in 2019 and 2020 compared to 2018 (Tables 4 and 5). In small plots, the amount of dead material on bracken plants was greater in 2019 and 2020 compared to 2018 (Figure 7d). The number of bracken plants in both small and large plots declined with time since fire (Figures 7e and 8e).

**TABLE 5:** Results from Bayesian generalized linear model analyzing whether *Pteridium esculentum* (bracken) morphology including: plant width, height to bottom frond, height to top, percentage of dead material on each plant, and number of plants in 200 m × 200 m plots, are influenced by herbivore access (open/partial/closed) and year (2018/19/20)

Coefficient	Width (cm)		Bottom Height (cm)		Top Height (cm)		Dead (%)		Count	
	Est.	CI (95%)	Est.	CI (95%)	Est.	CI (95%)	Est.	CI (95%)	Est.	CI (95%)
Intercept	**51.33**	**45.37, 56.53**	**25.73**	**20.58, 30.33**	**50.00**	**42.32, 57.03**	−0.22	−0.85, 0.43	**3.68**	**2.95, 4.41**
Herbivory (closed)	**−5.67**	**−9.85, −0.48**	0.22	−3.45, 4.58	−5.22	−11.41, 1.36	−0.03	−0.44, 0.37	0.17	−0.47, 0.85
Year (2019)	**9.62**	**6.22, 13.19**	**2.66**	**0.08, 5.23**	**11.66**	**8.42, 15.04**	0.19	−0.27, 0.64	**−0.2**	**−0.33, −0.07**
Year (2020)	**8.65**	**4.86, 12.43**	**7.33**	**4.77, 10.07**	**18.15**	**14.60, 21.79**	0.39	−0.07, 0.88	**−0.44**	**−0.59, −0.31**
Block B	**14.41**	**9.00, 20.84**	**8.18**	**3.09, 13.40**	**17.07**	**8.65, 26.01**	−0.17	−0.74, 0.39	0.76	−0.14, 1.66
Block C	2.27	−4.65, 8.94	2.05	−3.74, 7.86	−0.68	−9.68, 8.56	0.05	−0.58, 0.65	−0.26	−1.17, 0.72
Block D	4.48	−1.30, 10.75	−0.45	−5.87, 4.86	2.31	−6.48, 10.98	−0.06	−0.64, 0.50	0.64	−0.23, 1.52
*N*	8_plot_		8_plot_		8_plot_		8_plot_		8_plot_	
Observations	1257		1257		1257		1257		24	
Marginal *R* ^2^/Conditional *R* ^2^	.087/.090		.064/.065		.162/.175		.001/.001		.765/.767	

Note

Estimates and 95% credible intervals are shown for the most parsimonious models (by WAIC and model simplicity; see Table S2 for model selection table). Rows that are in bold indicate that credible intervals do not overlap zero. Reference states for comparisons were open plots sampled in 2018.

## 4 DISCUSSION

The interactive effect of herbivory and fire on vegetation structure, composition, and dynamics is important but often overlooked (Foster et al., 2020). We used a manipulative exclosure experiment to address the question: How does post‐fire herbivory alter understory plant diversity and dominant plant attributes in a coastal eucalypt forest? We found evidence that herbivore exclusion and time since fire, and their interaction altered the understory plant community. Our key findings were that: (1) herbivore activity was reduced by the exclusion fences, but there was no evidence of a time since fire effect; (2) the exclusion of herbivores generally resulted in a richer, more diverse, and more even vegetation community; (3) time since fire was the strongest driver of morphological changes in bracken, which dominated the understory; and (4) the influence of the previous prescribed burns had no apparent legacy effects on vegetation measures.

### 4.1 Post‐fire herbivore activity

Fencing treatments reduced herbivore activity in partially closed and closed plots, but there was no time since fire effect. A lack of a time since fire effect was surprising as the macropod species present within our study have previously shown a selective preference for recently burnt patches (Foster et al., 2015; Hradsky et al., 2017; Meers & Adams, 2003; Parkins et al., 2019; Southwell & Jarman, 1987). This result might be explained by the large size of the 2017 wildfire (1600 ha) compared with other studies that focused on smaller burns (31 ha, Southwell & Jarman, 1987; 1 ha, Meers & Adams, 2003; 226 ha, Dexter et al., 2013; 0.25 ha, Foster et al., 2015; and 714 ha, Hradsky et al., 2017). Macropods may be selecting for burnt patches within our study area but, due to the large size of the 2017 wildfire, increased occurrence may have been distributed over a large area, resulting in only a nuanced effect at our study plots. If fire size is impacting herbivore response, future prescribed burning practices may need to accommodate local herbivore density. To confirm this, more investigations would be required to determine the relationship between fire size and herbivore density.

### 4.2 Shifting vegetation communities

Knowledge of modifications to vegetation communities by large herbivores within fire‐prone ecosystems is increasing (Crowther et al., 2016; Tuft et al., 2012). We found that post‐fire herbivory reduced plant species richness, diversity, and evenness and increased plant dominance measures. This was expected as foraging pressure by large herbivores has been shown to decrease diversity and increase species dominance in other ecosystems (Bakker et al., 2006; Connor et al., 2021; Tuft et al., 2012). This finding confirms that increased foraging pressure from large herbivores following a wildfire also results in a more depauperate vegetation community, consistent with previous studies of smaller prescribed/experimental burns (Foster et al., 2015; Parkins et al., 2019).

Our study revealed that the richest and most diverse plant communities occurred directly after fire. These findings are consistent with earlier studies indicating richness decreases with increasing time since fire (Foster et al., 2018; Ross et al., 2002). Higher species richness immediately after fire was expected in eucalypt forest understory as fire typically stimulates new growth and the germination of seedlings, and many species can re‐sprout after fire (Dixon et al., 2018). Furthermore, within large plots, we observed that with increasing time since fire, herbivore exclusion promoted a more diverse and more even community. Preventing large herbivores from accessing burnt patches may therefore promote a richer and more diverse understory.

### 4.3 Morphology of bracken

The increase in *P*. *esculentum* dominance within Booderee National Park has been an ongoing concern for resource managers (Dexter et al., 2013). However, this study did not uncover evidence of differences in bracken morphology with altered herbivore activity. This was surprising as we expected this unpalatable plant species to thrive in plots accessible to macropods where increased foraging of palatable plants would reduce interspecies competition with bracken (Archibald & Hempson, 2016). Previous research indicated high plant diversity in understory forest communities can positively affect the biomass and survivability of all plants (Cook‐Patton et al., 2014). It is possible that bracken is experiencing a win–win scenario in post‐fire landscapes, whether browsed or unbrowsed. Increased herbivory may be reducing interspecies competition by decreasing species diversity, promoting the dominance of bracken. Conversely, reduced herbivory may be accommodating positive interspecific interactions from increased species diversity, resulting in healthier bracken plants.

Our findings indicate that increasing time since fire led to the reduced abundance and altered physical attributes of bracken plants. The amount of dead material on bracken increased with increasing time since fire in small plots. This dead material may contribute directly to fire risk by increasing potential fire behavior (Cheney et al., 2012). However, no changes were observed within the large plots. Potential differences in our results may be due to the difference between time since exclosure (9 years vs. 3 years) or the time to establishing the larger manipulative experiment following the wildfire in 2017 (10 months). The latter effect may have allowed macropods to modify the understory before the fences affected herbivore activity, reducing the contrast between plots. Extending the survey to future years may reveal important findings as dead biomass of bracken usually peaks at 4 to 7 years following disturbance (Bray, 1991; Parkins et al., 2019). Longer‐term datasets (>3 years) may be required to document post‐fire bracken growth within forested communities.

### 4.4 Fire history

We found no evidence that previous prescribed burns in 2012 influenced herbivore activity, vegetation community measures, or bracken morphology following the 2017 wildfire. This was surprising as repeated fires at short intervals (5 years in our study) have been observed to drive large herbivores to suppress palatable plants, locally decreasing plant diversity, while promoting the dominance of unpalatable, fire‐resistant plants like bracken (Archibald & Hempson, 2016; Pietrzykowski et al., 2003; Wyse et al., 2016). This may be because of strong effects of the 2017 fire and the herbivore masked any remaining effects on vegetation of the 2012 fires. It is possible that effects of the short fire interval may become apparent with increasing time since fire (i.e., reduced effect of 2017 fire), where impacts on slower growing species, and in particular obligate seeding shrub species, become easier to detect.

## 5 CONCLUSION

Our study highlights the impacts of post‐fire herbivory on vegetation communities. Post‐fire herbivory decreases richness, diversity, and evenness measures and increases the dominance of few species leading to a more depauperate vegetation community. Future management of forest ecosystems should account for local populations of large herbivores. Increased understory plant diversity may be achieved by reducing herbivore numbers or preventing access to burnt patches following fire. Conversely, where herbivore impacts align with management goals (e.g., control of palatable weeds, or reducing fuel biomass for fire hazard reduction), small prescribed burns may be effective in concentrating foraging pressure by herbivores in target areas.

## CONFLICTS OF INTEREST

The authors declare no conflicts of interest.

## AUTHOR CONTRIBUTIONS

**Matthew Chard:** Conceptualization (lead); Data curation (lead); Formal analysis (lead); Investigation (lead); Methodology (lead); Project administration (lead); Resources (lead); Validation (lead); Visualization (lead); Writing – original draft (lead); Writing – review & editing (lead). **Claire N Foster:** Conceptualization (supporting); Formal analysis (supporting); Funding acquisition (supporting); Investigation (supporting); Methodology (supporting); Supervision (lead); Writing – review & editing (equal). **David B Lindenmayer:** Conceptualization (supporting); Funding acquisition (supporting); Resources (equal); Supervision (equal); Visualization (supporting); Writing – review & editing (equal). **Geoffrey J Cary:** Conceptualization (supporting); Methodology (supporting); Supervision (supporting); Writing – review & editing (equal). **Christopher MacGregor:** Data curation (supporting); Investigation (supporting); Project administration (supporting); Resources (supporting); Supervision (supporting); Writing – review & editing (supporting). **Wade Blanchard:** Formal analysis (supporting); Validation (supporting); Writing – review & editing (supporting).

### OPEN RESEARCH BADGES

This article has earned an Open Data Badge for making publicly available the digitally‐shareable data necessary to reproduce the reported results. The data is available at https://datadryad.org/stash/share/PRjYHam6K3TZZ‐9‐ZDOc96p1qNzDOH8yXz196j1RLuI.

## Supporting information

Table S1‐S3

## Data Availability

The data that support the findings of this study are openly available in Dryad using the digital object identifier (DOI): https://doi.org/10.5061/dryad.dr7sqvb0r.
