# Corrigendum

**DOI:** 10.1002/ece3.9061

**Published:** 2022-07-05

In the recent article by Chard et al. (2022), the last author in reference, Foster et al. (2016), is incorrect. It should read as follow:

Foster, C. N., Barton, P. S., Sato, C. F., Wood, J. T., MacGregor, C. I. & Lindenmayer, D. B. (2016). Herbivory and fire interact to affect forest understory habitat, but not its use by small vertebrates. *Animal Conservation*, 19(1), 15–25. https://doi.org/10.1111/acv.12210

The authors apologize for the error.
