# Supplementary material for: Post‐fire pickings: Large herbivores alter understory vegetation communities in a coastal eucalypt forest
Source: Ecol Evol. 2022 Apr 23;12(4):e8828. doi: 10.1002/ece3.8828 (PMC9034452; doi:10.1002/ece3.8828)
Supplement: Supplementary file 1 — Table S1‐S3 [file ECE3-12-e8828-s001.docx]

**APPENDIX**

Table S1: Model selection results from an analysis of herbivory (open/partial/closed), fire (unburnt/burnt), and time since fire (TSF, continuous) influences on macropod scats. Bayesian generalized linear models were constructed with negative binomial distribution. Within all models, season and block were included as fixed effects and plot was included as a random effect. Results are ranked based of WAIC for small (25 m x 25 m) and large (200 m x 200 m) plots. The most parsimonious model, indicated in bold, was selected from the simplest (ie. least variables) model under the WAIC threshold of 2 (indicated by dashed line).

|  | **SMALL PLOTS** |  |  |
| --- | --- | --- | --- |
| **Model** | **Model Description** | **WAIC** | **SE** |
| **4** | Herbivory+Fire+TSF + Herbivory:TSF | 0.00 | 0.00 |
| **1** | **Herbivory+Fire+TSF** | **0.30** | **3.34** |
| **6** | Herbivory+Fire+TSF + Herbivory:Fire + Herbivory:TSF | 0.35 | 2.20 |
| **2** | Herbivory+Fire+TSF + Herbivory:Fire | 1.34 | 3.94 |
| **3** | Herbivory+Fire+TSF + Fire:TSF | 1.44 | 3.54 |
| **7** | Herbivory+Fire+TSF + Fire:TSF + Herbivory:TSF | 1.69 | 1.26 |
| **8** | Herbivory+Fire+TSF + Herbivory:Fire+ Herbivory:TSF + Fire:TSF | 1.74 | 2.64 |
| **5** | Herbivory+Fire+TSF + Herbivory:Fire+ Fire:TSF | 1.90 | 3.98 |
| **9** | Herbivory+Fire+TSF + Herbivory:Fire+ Herbivory:TSF + Fire:TSF + Herbivory:Fire:TSF | 1.98 | 4.33 |
|  | **LARGE PLOTS** |  |  |
| **Model** | **Model Description** | **WAIC** | **SE** |
| **1** | **Herbivory+TSF** | **0.00** | **0.00** |
| **2** | Herbivory+TSF + Herbivory:TSF | 0.77 | 2.40 |

Table S2: Model selection results from an analysis of herbivory (open/partial/closed), fire (unburnt/burnt), and time since fire (TSF, continuous) influences on vegetation community measures. Plant community measures were species richness, diversity (Simpson’s reciprocal index, 1/D), evenness (Simpson’s evenness, E_1/D_) and dominance (Berger- Parker, d). Bayesian generalized linear models were constructed with Poisson, for species richness, or Gaussian distribution. Within all models, block was included as a fixed effect and plot was included as a random effect. Results are ranked based of WAIC for small (25 m x 25 m) and large (200 m x 200 m) plots. The most parsimonious model, indicated in bold, was selected from the simplest (ie. least variables) model under the WAIC threshold of 2 (indicated by dashed line).

|  | **SMALL PLOTS** |  |  |
| --- | --- | --- | --- |
| **Model** | **Model Description** | **WAIC** | **SE** |
|  | *Species Richness* |  |  |
| **4** | Herbivory+Fire+TSF + Herbivory:TSF | 0.00 | 0.00 |
| **6** | Herbivory+Fire+TSF + Herbivory:Fire + Herbivory:TSF | 0.16 | 0.91 |
| **1** | **Herbivory+Fire+TSF** | **0.73** | **3.40** |
| **2** | Herbivory+Fire+TSF + Herbivory:Fire | 0.79 | 3.50 |
| **7** | Herbivory+Fire+TSF + Fire:TSF + Herbivory:TSF | 2.06 | 1.21 |
| **8** | Herbivory+Fire+TSF + Herbivory:Fire + Herbivory:TSF + Fire:TSF | 2.70 | 1.63 |
| **3** | Herbivory+Fire+TSF + Fire:TSF | 2.75 | 3.72 |
| **5** | Herbivory+Fire+TSF + Herbivory:Fire+ Fire:TSF | 3.32 | 3.85 |
| **9** | Herbivory+Fire+TSF + Herbivory:Fire + Herbivory:TSF + Fire:TSF + Herbivory:Fire:TSF | 5.86 | 2.62 |
|  |  |  |  |
|  | *Simpson’s Diversity* |  |  |
| **6** | Herbivory+Fire+TSF + Herbivory:Fire + Herbivory:TSF | 0.00 | 0.00 |
| **4** | **Herbivory+Fire+TSF + Herbivory:TSF** | **0.28** | **2.04** |
| **7** | Herbivory+Fire+TSF + Fire:TSF + Herbivory:TSF | 2.65 | 2.93 |
| **8** | Herbivory+Fire+TSF + Herbivory:Fire + Herbivory:TSF + Fire:TSF | 3.41 | 2.01 |
| **1** | Herbivory+Fire+TSF | 4.14 | 7.67 |
| **2** | Herbivory+Fire+TSF + Herbivory:Fire | 4.59 | 6.90 |
| **9** | Herbivory+Fire+TSF + Herbivory:Fire + Herbivory:TSF + Fire:TSF + Herbivory:Fire:TSF | 5.78 | 4.97 |
| **3** | Herbivory+Fire+TSF + Fire:TSF | 7.76 | 7.36 |
| **5** | Herbivory+Fire+TSF + Herbivory:Fire+ Fire:TSF | 7.82 | 7.99 |
|  |  |  |  |
|  | *Shannon’s Evenness* |  |  |
| **1** | **Herbivory+Fire+TSF** | **0.00** | **0.00** |
| **2** | Herbivory+Fire+TSF + Herbivory:Fire | 0.26 | 1.86 |
| **4** | Herbivory+Fire+TSF + Herbivory:TSF | 0.77 | 5.16 |
| **6** | Herbivory+Fire+TSF + Herbivory:Fire + Herbivory:TSF | 1.13 | 5.60 |
| **3** | Herbivory+Fire+TSF + Fire:TSF | 2.41 | 2.54 |
| **5** | Herbivory+Fire+TSF + Herbivory:Fire+ Fire:TSF | 2.52 | 3.08 |
| **7** | Herbivory+Fire+TSF + Fire:TSF + Herbivory:TSF | 3.89 | 5.48 |
| **8** | Herbivory+Fire+TSF + Herbivory:Fire + Herbivory:TSF + Fire:TSF | 4.30 | 5.90 |
| **9** | Herbivory+Fire+TSF + Herbivory:Fire + Herbivory:TSF + Fire:TSF + Herbivory:Fire:TSF | 6.88 | 7.74 |
|  |  |  |  |
|  | *Berger-Parker Dominance* |  |  |
| **8** | Herbivory+Fire+TSF + Herbivory:Fire + Herbivory:TSF + Fire:TSF | 0.00 | 0.00 |
| **7** | Herbivory+Fire+TSF + Fire:TSF + Herbivory:TSF | 0.28 | 2.08 |
| **4** | **Herbivory+Fire+TSF + Herbivory:TSF** | **1.12** | **4.87** |
| **6** | Herbivory+Fire+TSF + Herbivory:Fire + Herbivory:TSF | 1.16 | 4.22 |
| **9** | Herbivory+Fire+TSF + Herbivory:Fire + Herbivory:TSF + Fire:TSF + Herbivory:Fire:TSF | 2.04 | 4.79 |
| **1** | Herbivory+Fire+TSF | 6.64 | 7.09 |
| **2** | Herbivory+Fire+TSF + Herbivory:Fire | 6.69 | 8.48 |
| **3** | Herbivory+Fire+TSF + Fire:TSF | 6.70 | 7.91 |
| **5** | Herbivory+Fire+TSF + Herbivory:Fire+ Fire:TSF | 8.10 | 7.67 |
|  |  |  |  |
|  | **LARGE PLOTS** |  |  |
| **Model** | **Model Description** | **WAIC** | **SE** |
|  | *Species Richness* |  |  |
| **2** | **Herbivory+TSF + Herbivory:TSF** | **0.00** | **0.00** |
| **1** | Herbivory+TSF | 2.21 | 2.78 |
|  |  |  |  |
|  | *Simpson’s Diversity* |  |  |
| **2** | **Herbivory+TSF + Herbivory:TSF** | **0.00** | **0.00** |
| **1** | Herbivory+TSF | 3.08 | 7.14 |
|  |  |  |  |
|  | *Shannon’s Evenness* |  |  |
| **2** | **Herbivory+TSF + Herbivory:TSF** | **0.00** | **0.00** |
| **1** | Herbivory+TSF | 8.98 | 7.06 |
|  |  |  |  |
|  | *Berger-Parker Dominance* |  |  |
| **1** | **Herbivory+TSF** | **0.00** | **0.00** |
| **2** | Herbivory+TSF + Herbivory:TSF | 1.89 | 3.54 |

Table S3: Model selection results from an analysis of herbivory (open/partial/closed), fire (unburnt/burnt), and time since fire (TSF, continuous) influences on morphological measurements of the dominant understory bracken (Pteridium esculentum). Morphological responses include width, height to bottom frond, top height, percent of dead material and number of plants. Bayesian generalized linear models were constructed with Poisson (for count data), zero-one-inflated (for percent data) or Gaussian distribution. Within all models, block was included as a fixed effect and plot was included as a random effect. Results are ranked based of WAIC for small (25 m x 25 m) and large (200 m x 200 m) plots. The most parsimonious model, indicated in bold, was selected from the simplest (ie. least variables) model under the WAIC threshold of 2 (indicated by dashed line).

|  | **SMALL PLOTS** |  |  |
| --- | --- | --- | --- |
| **Model** | **Model Description** | **WAIC** | **SE** |
|  | *Width (cm)* |  |  |
| **1** | **Herbivory+Fire+TSF** | **0.00** | **0.00** |
| **4** | Herbivory+Fire+TSF + Herbivory:TSF | 0.26 | 2.31 |
| **6** | Herbivory+Fire+TSF + Herbivory:Fire + Herbivory:TSF | 0.31 | 2.36 |
| **3** | Herbivory+Fire+TSF + Fire:TSF | 0.40 | 1.36 |
| **2** | Herbivory+Fire+TSF + Herbivory:Fire | 0.59 | 2.88 |
| **9** | Herbivory+Fire+TSF + Herbivory:Fire + Herbivory:TSF + Fire:TSF + Herbivory:Fire:TSF | 0.64 | 0.12 |
| **7** | Herbivory+Fire+TSF + Fire:TSF + Herbivory:TSF | 1.05 | 2.74 |
| **8** | Herbivory+Fire+TSF + Herbivory:Fire + Herbivory:TSF + Fire:TSF | 1.18 | 2.75 |
| **5** | Herbivory+Fire+TSF + Herbivory:Fire+ Fire:TSF | 1.39 | 1.42 |
|  |  |  |  |
|  | *Bottom Height (cm)* |  |  |
| **4** | **Herbivory+Fire+TSF + Herbivory:TSF** | **0.00** | **0.00** |
| **6** | Herbivory+Fire+TSF + Herbivory:Fire + Herbivory:TSF | 0.34 | 0.26 |
| **7** | Herbivory+Fire+TSF + Fire:TSF + Herbivory:TSF | 1.50 | 0.66 |
| **8** | Herbivory+Fire+TSF + Herbivory:Fire + Herbivory:TSF + Fire:TSF | 1.67 | 0.66 |
| **1** | Herbivory+Fire+TSF | 3.01 | 4.34 |
| **2** | Herbivory+Fire+TSF + Herbivory:Fire | 3.46 | 1.54 |
| **9** | Herbivory+Fire+TSF + Herbivory:Fire + Herbivory:TSF + Fire:TSF + Herbivory:Fire:TSF | 3.51 | 4.31 |
| **3** | Herbivory+Fire+TSF + Fire:TSF | 4.46 | 4.34 |
| **5** | Herbivory+Fire+TSF + Herbivory:Fire+ Fire:TSF | 4.90 | 4.31 |
|  |  |  |  |
|  | *Top Height (cm)* |  |  |
| **1** | **Herbivory+Fire+TSF** | **0.00** | **0.00** |
| **2** | Herbivory+Fire+TSF + Herbivory:Fire | 0.58 | 0.15 |
| **3** | Herbivory+Fire+TSF + Fire:TSF | 2.15 | 1.60 |
| **5** | Herbivory+Fire+TSF + Herbivory:Fire+ Fire:TSF | 2.75 | 1.57 |
| **4** | Herbivory+Fire+TSF + Herbivory:TSF | 3.30 | 1.93 |
| **6** | Herbivory+Fire+TSF + Herbivory:Fire + Herbivory:TSF | 3.74 | 1.94 |
| **7** | Herbivory+Fire+TSF + Fire:TSF + Herbivory:TSF | 5.42 | 2.56 |
| **8** | Herbivory+Fire+TSF + Herbivory:Fire + Herbivory:TSF + Fire:TSF | 5.66 | 2.54 |
| **9** | Herbivory+Fire+TSF + Herbivory:Fire + Herbivory:TSF + Fire:TSF + Herbivory:Fire:TSF | 6.91 | 2.82 |
|  |  |  |  |
|  | *Dead (%)* |  |  |
| **1** | **Herbivory+Fire+TSF** | **0.00** | **0.00** |
| **3** | Herbivory+Fire+TSF + Fire:TSF | 2.69 | 2.31 |
| **2** | Herbivory+Fire+TSF + Herbivory:Fire | 3.92 | 0.66 |
| **4** | Herbivory+Fire+TSF + Herbivory:TSF | 5.47 | 2.45 |
| **5** | Herbivory+Fire+TSF + Herbivory:Fire+ Fire:TSF | 6.62 | 2.39 |
| **6** | Herbivory+Fire+TSF + Herbivory:Fire + Herbivory:TSF | 7.58 | 3.64 |
| **7** | Herbivory+Fire+TSF + Fire:TSF + Herbivory:TSF | 8.94 | 2.67 |
| **8** | Herbivory+Fire+TSF + Herbivory:Fire + Herbivory:TSF + Fire:TSF | 10.38 | 6.02 |
| **9** | Herbivory+Fire+TSF + Herbivory:Fire + Herbivory:TSF + Fire:TSF + Herbivory:Fire:TSF | 10.85 | 3.89 |
|  |  |  |  |
|  | *Count* |  |  |
| **7** | Herbivory+Fire+TSF + Fire:TSF + Herbivory:TSF | 0.00 | 0.00 |
| **6** | Herbivory+Fire+TSF + Herbivory:Fire + Herbivory:TSF | 1.31 | 5.80 |
| **8** | Herbivory+Fire+TSF + Herbivory:Fire + Herbivory:TSF + Fire:TSF | 1.56 | 1.90 |
| **4** | **Herbivory+Fire+TSF + Herbivory:TSF** | **1.73** | **5.20** |
| **9** | Herbivory+Fire+TSF + Herbivory:Fire + Herbivory:TSF + Fire:TSF + Herbivory:Fire:TSF | 2.72 | 5.39 |
| **3** | Herbivory+Fire+TSF + Fire:TSF | 11.46 | 11.97 |
| **5** | Herbivory+Fire+TSF + Herbivory:Fire+ Fire:TSF | 12.80 | 11.99 |
| **1** | Herbivory+Fire+TSF | 14.52 | 16.59 |
| **2** | Herbivory+Fire+TSF + Herbivory:Fire | 15.70 | 16.67 |
|  |  |  |  |
|  | **LARGE PLOTS** |  |  |
| **Model** | **Model Description** | **WAIC** | **SE** |
|  | *Width (cm)* |  |  |
| **1** | **Herbivory+TSF** | **0.00** | **0.00** |
| **2** | Herbivory+TSF + Herbivory:TSF | 3.79 | 1.96 |
|  |  |  |  |
|  | *Bottom Height (cm)* |  |  |
| **1** | **Herbivory+TSF** | **0.00** | **0.00** |
| **2** | Herbivory+TSF + Herbivory:TSF | 3.53 | 2.49 |
|  |  |  |  |
|  | *Top Height (cm)* |  |  |
| **1** | **Herbivory+TSF** | **0.00** | **0.00** |
| **2** | Herbivory+TSF + Herbivory:TSF | 3.22 | 2.53 |
|  |  |  |  |
|  | *Dead (%)* |  |  |
| **1** | **Herbivory+TSF** | **0.00** | **0.00** |
| **2** | Herbivory+TSF + Herbivory:TSF | 3.41 | 2.10 |
|  |  |  |  |
|  | *Count* |  |  |
| **1** | **Herbivory+TSF** | **0.00** | **0.00** |
| **2** | Herbivory+TSF + Herbivory:TSF | 3.90 | 0.76 |
